# Delay to surgical treatment in lung cancer patients and its impact on survival in a video-assisted thoracoscopic lobectomy cohort

**DOI:** 10.1038/s41598-021-84162-4

**Published:** 2021-03-01

**Authors:** Florian Ponholzer, Veronika Kroepfl, Caecilia Ng, Herbert Maier, Florian Kocher, Paolo Lucciarini, Dietmar Öfner, Florian Augustin

**Affiliations:** 1 Department of Visceral, Transplant and Thoracic Surgery, Medical University Innsbruck, Anichstrasse 35, 6020 Innsbruck, Austria; 2 Department of Internal Medicine V: Hematology and Oncology, Medical University Innsbruck, Innsbruck, Austria

**Keywords:** Oncology, Surgical oncology

## Abstract

Patient pathways from first suspicious imaging until final surgical treatment vary and in some instances cause considerable delay. This study aims to investigate the impact of this delay on survival of lung cancer patients. The institutional database was queried to identify patients with primary lung cancer who were treated with primary surgery. Time intervals were defined as date of first suspicious medical images until date of surgical treatment. All patients received PET-CT staging and tissue confirmation prior to treatment planning in a multidisciplinary tumor board. Patients with unknown date of first contact, follow-up CT-scans of pulmonary nodules, or neoadjuvant therapy were excluded. In total, 287 patients treated between 2009 and 2017 were included for further analysis. Median time between first suspicious medical imaging and surgical therapy was 62 (range 23–120) days and did not differ between male and female patients. Patients were then classified into two groups according to the duration of the medical work-up: group A up to 60 days, and group B from 61 to 120 days. Clinical T and N stages were comparable between the groups. There was no difference in overall survival between the two groups. In the subgroup of cT2 tumors (87 patients), there was a significant survival benefit for patients in group A (*p* = 0.043), while nodal stages, stage migration, lymphatic vessel invasion, grading and other potentially survival-influencing clinical parameters were comparable between the groups. Delay between diagnosis and treatment of lung cancer may result in dismal outcome. Efforts need to focus on improving and streamlining patient pathways to shorten the delay until surgical treatment to a minimum. Process improvement might be achieved by stringent interdisciplinary work-up and a patient-centered approach.

## Introduction

Lung cancer is the most common cancer worldwide with more than 1.8 million newly diagnosed patients in 2012, nearly half of the diagnoses occur in first world countries North America, Europe, Australia, New Zealand and Japan^1^.

The pathway from diagnosis to treatment can be a complex and time-consuming process as it involves many different specialties ranging from primary health physicians to radiologists, surgeons, oncologists and radiotherapists. In this complex interplay, time from diagnosis to surgical treatment may last for up to months. Often delays in diagnosis and treatment are associated with physical distress in tumour-patients^2^. As Robinson et al. and Christensen et al. have described earlier, there is a relationship between longer symptom-to –diagnosis interval (SDI) and higher cancer staging^3,4^. Another problem is the waiting period from diagnosis to final surgical treatment. Often there is an imbalance between health care systems supply and the required medical services for further diagnostics. Moreover, inefficient coordination between professional healthcare supplies adds further friction to this process.

The aim of our study was to determine the time from first suspicious imaging to primary surgical treatment in lung cancer patients and its impact on oncological outcome and survival.

## Materials and methods

### Patient selection

**Figure 1:**
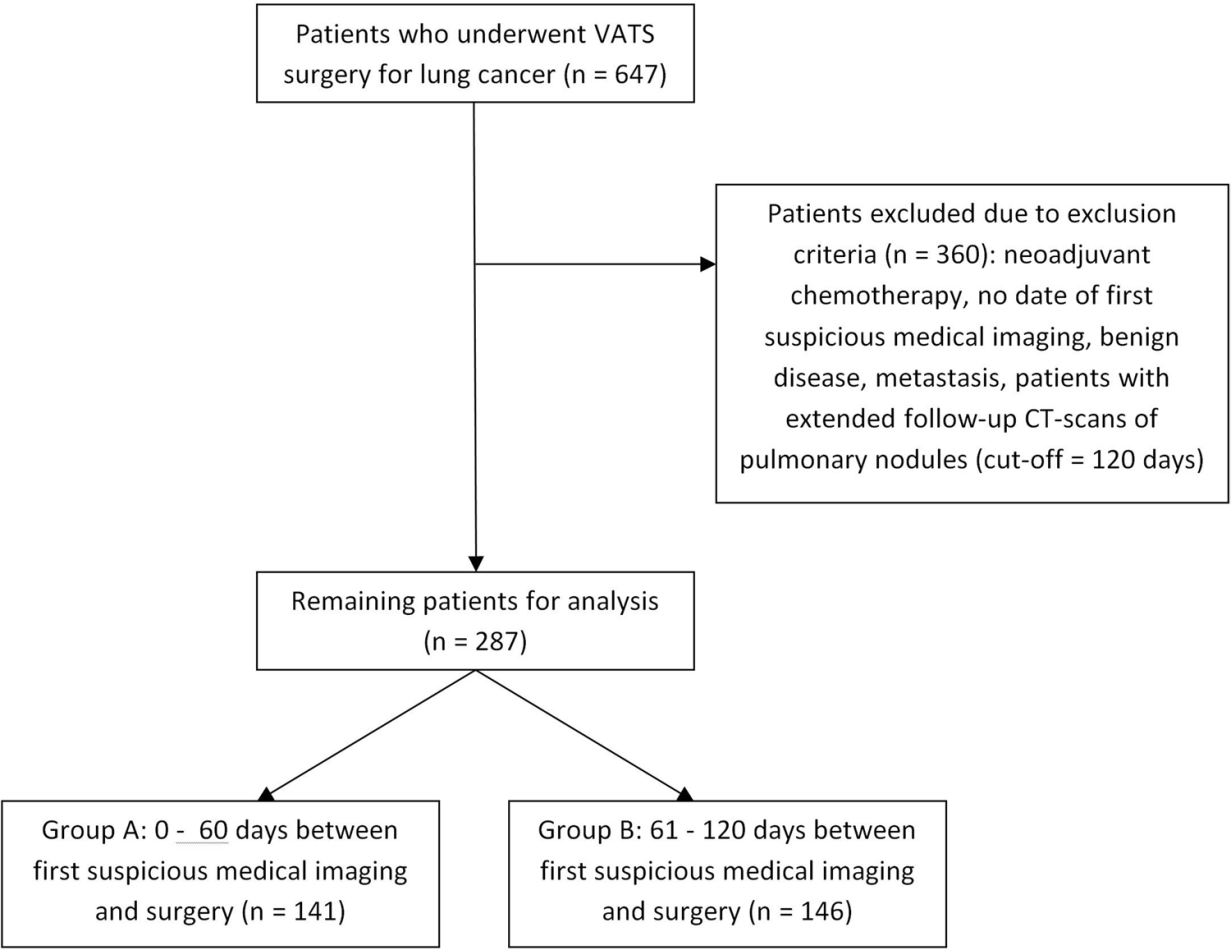
CONSORT diagram of patient selection for statistical analysis.

All patients admitted for anatomical VATS resection for primary lung cancer at a single center surgical institution were studied retrospectively. Authorization for data collection was granted by the local ethics committee (Ethikkommission der Medizinischen Universität Innsbruck). All research was performed in accordance with the relevant guidelines and regulations; individual informed consent was waived by the ethics committee due to the retrospective nature of the study. Between February 2009 and October 2017 a total of 647 patients were included in the database. Patients with neoadjuvant chemotherapy, no date for the first suspicious medical imaging, benign disease, metastasis or extended follow-up computed tomography (CT) scans of pulmonary nodules (cut-off = 120 days) were excluded. A cut-off value of 120 days was chosen to exclude patients with an extended follow up regime, which may artificially raise the delay to surgery. If a patient’s data did not mention an exact date for the first suspicious medical imaging, the patient was excluded from analysis. In total, 287 patients were included in the final dataset. (Fig. 1).

### Definitions

The first suspicious medical imaging was defined as the first X-Ray, CT scan or positron emission tomography (PET), in which a pulmonary nodule is mentioned and is advised for further examination according to the guidelines of the Fleischner Society.

Treatment was defined as the date of primary anatomical VATS resection.

Overall survival was defined as the time from date of surgery until date of last recorded follow up or patient´s death.

Patients were grouped according to the duration from first suspicious imaging until surgery: group A with patients, who received surgery within 60 days after the first suspicious medical imaging and group B with patients, who received surgery 60 to 120 days after their first suspicious medical imaging.

The patient pathway from the first suspicious medical imaging to primary VATS surgery included a CT scan, PET and pathological workup for all patients. Further diagnostic modalities like transthoracic needle aspiration (TTNA) or bronchoscopy varied in the study population.

For tumour staging the seventh edition of the UICC “TNM classification of malignant tumours” has been used^5^.

### Statistical analysis

Statistical analysis was performed with IBM SPSS Statistics 24 (IBM Corporation, Armonk, NY, USA).

Pearson’s chi-squared test was used for identifying correlations between categorical variables. The Kaplan–Meier estimator was used to analyze the overall survival between group A and B and their respective subgroups. The Breslow test was performed to analyze the significance of overall survival differences between samples. T-Test was used to compare the mean delay to receive surgery and the mean age between for the male and female study population. Logistic regression analysis was used to calculate odds ratios. Statistical significance was assumed for a p-value < 0.05.

## Results

A total of 287 patients were analyzed. To evaluate the impact of delay to surgical treatment, patients were split into two groups: Group A with 141 out of 287 patients (49.1%), who received surgery within 60 days and Group B with 146 patients (50.9%), who received surgery within 61 to 120 days. The median delay in the dataset was 62 (range 23 – 120) days. Patients’ characteristics are displayed in Table 1.

**Table 1:** Patients’ demographics.

Factor*	Group A (0–60 days)	Group B (61–120 days)	*p* Value
Age (years), median (range)	64 (18—83)	66 (31—84)	0.152
Gender (%)			0.477
Female	60 (42.6)	69 (47.3)	
Male	81 (57.4)	77 (52.7)	
Histology (%)			0.372
Adenocarcinoma	89 (63.1)	102 (69.9)	
Squamous-cell carcinoma	27 (19.1)	28 (19.2)	
Large cell cancer	7 (5.0)	3 (2.1)	
Others	18 (12.8)	13 (8.9)	
Clinical UICC stage (%)			0.605
IA	72 (51.4)	76 (53.1)	
IB	18 (12.9)	17 (11.9)	
IIA	29 (20.7)	20 (14.0)	
IIB	13 (9.3)	17 (11.9)	
IIIA	8 (5.7)	11 (7.7)	
IIIB	0	1 (0.7)	
IV	0	1^a^ (0.7)	
Pathological UICC stage (%)			0.719
IA	66 (46.8)	73 (50.0)	
IB	23 (16.3)	17 (11.6)	
IIA	23 (16.3)	21 (14.4)	
IIB	12 (8.5)	17 (11.6)	
IIIA	17 (12.1)	17 (11.6)	
IIIB	0	0	
IV	0	1^a^ (0.7)	
Grading			0.213
1	10 (7.8)	11 (8.1)	
2	61 (47.3)	78 (57.4)	
3	58 (45.0)	47 (34.6)	
Median delay to surgery (range)	47 (23–60)	82 (61–120)	
Modality of tissue confirmation (%)			0.002
Mediastinoscopy, EBUS or bronchoscopy	15 (10.6)	5 (3.4)	
CT-guided Biopsy	112 (79.4)	108 (74.0)	
Both modalities	14 (9.9)	32 (21.9)	
No (or inconclusive) tissue confirmation	0	1 (0.7)	
PET-CT (%)	141 (100)	146 (100)	

^a^65-year old cT2bN0M1 patient with one PET-positive T11 vertebral metastasis. Tumour board decided for primary VATS resection and radiotherapy of the metastasis.

*Some factors might not add up to 100% as data might have been inconclusive or not available (e.g. no grading in the pathological report, inconclusive preoperative staging, missing data from the primary care sector etc).

All patients received PET-CT staging and most of the patients also received attempts of tissue confirmation, 92.7% through CT-guided biopsy, 23.0% through mediastinoscopy, endobronchial ultrasound (EBUS) or bronchoscopy and 16.0% received both modalities. In accordance with a multidisciplinary tumour-board one patient did not receive any modality of tissue confirmation, because of dual antiplatelet therapy and radiologically distinct findings. All patients were discussed in a multidisciplinary tumour-board.

### Survival

**Figure 2:**
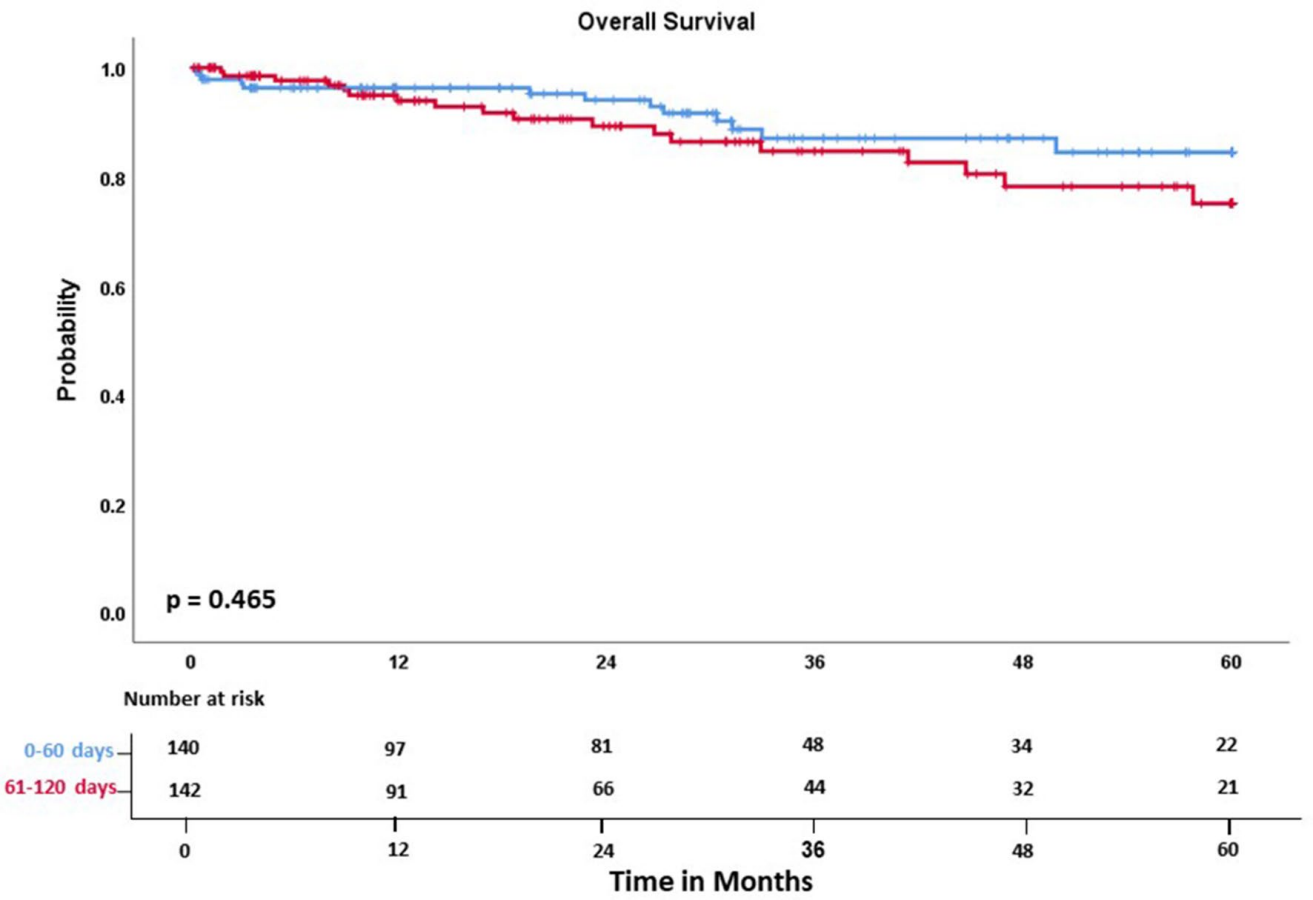
Overall survival for all patients did not show any difference between group A and B.

Two-year and 5-year overall survival between Group A and B did not differ significantly (94.1% vs. 89.3%, *p* = 0.469; 84.5% vs. 75.1%, *p* = 0.465, Fig. 2).

**Figure 3:**
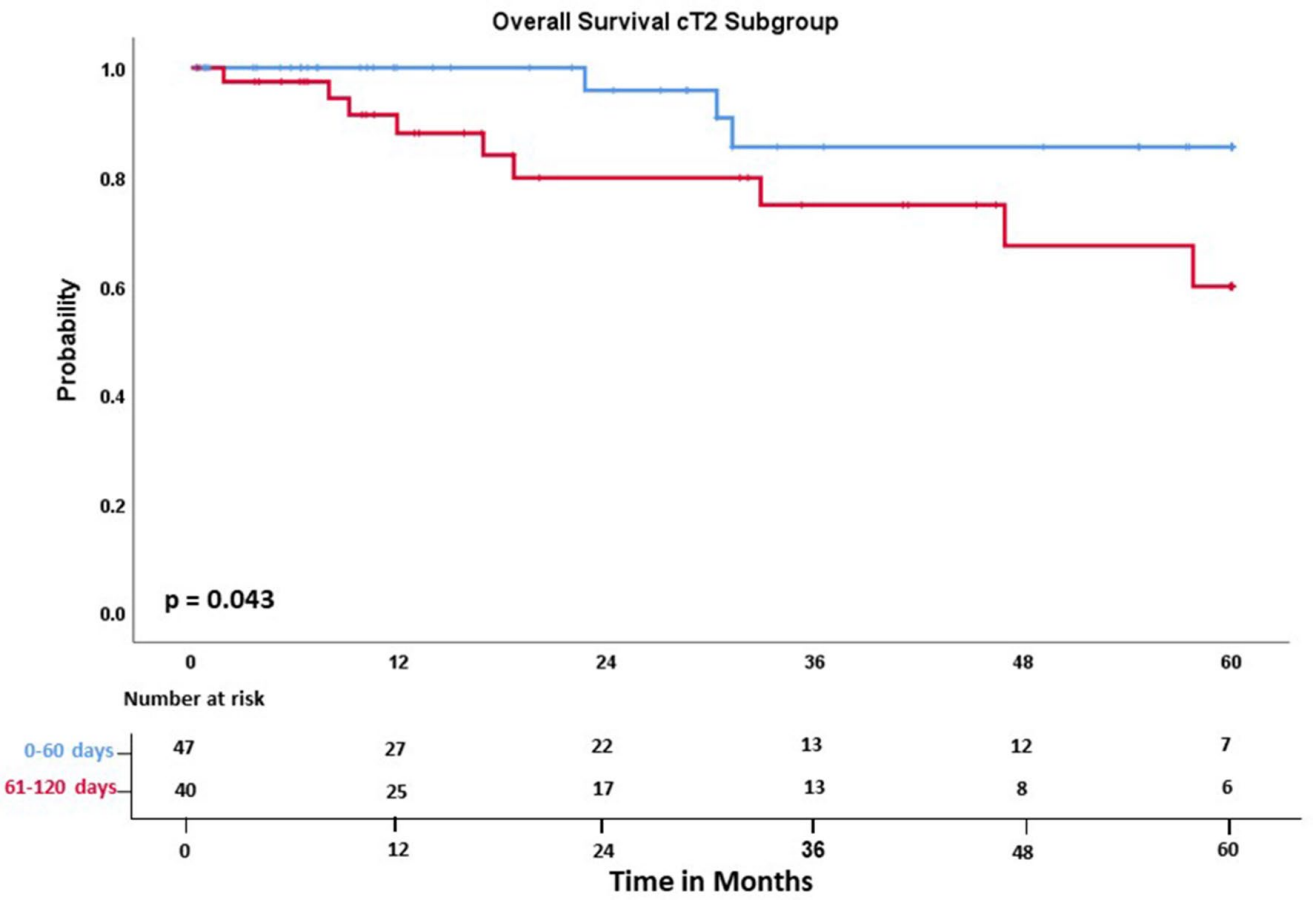
There was a significant survival benefit for cT2 patients with in group A.

When subgroups, as seen in Table 2 and 3, were analyzed separately, cT2-staged patients showed a significant 5-year overall survival benefit, when receiving surgery in 60 days or less (85.4% vs. 59.8%; *p* = 0.043, Fig. 3). Within this subgroup, there was no statistical difference regarding gender distribution, clinical nodal status, or pathologic nodal status between group A and B. There was also no difference in nodal stage migration between group A and group B (upstaging in 6/45 patients in group A and 3/40 patients in group B, *p* = 0.491). There was also no difference in the cT2-subgroup when comparing the rate of invasion into lymphatic vessels between group A and group B (37.5% vs. 35.0%; *p* = 0.828).

**Table 2:** Patients´ characteristics – cT1 subgroup.

Factor	cT1 Group A (n = 82)	cT1 Group B (n = 83)	*p* Value
Age (years), median (range)	62 (18—83)	66 (39—80)	0.126
Gender (%)			0.351
Female	36 (43.9)	43 (51.8)	
Male	46 (56.1)	40 (48.2)	
Histology (%)			0.117
Adenocarcinoma	52 (63.4)	62 (74.7)	
Squamous-cell carcinoma	12 (14.6)	14 (16.9)	
Large cell cancer	5 (6.1)	2 (2.4)	
Others	13 (15.9)	5 (6.0)	
Tumour diameter (mm)	20.54	18.53	0.092
Clinical nodal status (%)			1
Negative	70 (85.4)	71 (86.6)	
Positive	12 (14.6)	11 (13.4)	
Pathological nodal status (%)			0.426
Negative	65 (79.3)	70 (84.3)	
Positive	17 (20.7)	13 (15.7)	
Grading (%)			0.911
1	7 (9.6)	7 (9.0)	
2	41 (56.2)	47 (60.3)	
3	25 (34.2)	24 (30.8)	
Nodal stage migration (%)			1
Negative to positive	9 (11.0)	9 (11.0)	
Lymphatic vessel invasion (%)			0.844
No invasion	65 (79.3)	66 (81.5)	
Invasion	17 (20.7)	15 (18.5)	
Tumour localisation (%)			0.286
Central	15 (18.3)	10 (12.0)	
Peripheral	67 (81.7)	73 (88.0)	
Modality of tissue confirmation (%)			0.033
Mediastinoscopy, EBUS or bronchoscopy	6 (7.3)	2 (2.4)	
CT-guided biopsy	73 (89.0)	70 (84.3)	
Both modalities	3 (3.7)	11 (13.3)	
No (or inconclusive) tissue confirmation	0	0

**Table 3:** Patients´ characteristics – cT2 subgroup.

Factor	cT2 Group A (n = 48)	cT2 Group B (n = 41)	*p* Value
Age (years), median (range)	66 (26—81)	66 (36—84)	0.933
Gender (%)			0.829
Female	19 (39.6)	15 (36.6)	
Male	29 (60.4)	26 (63.4)	
Histology (%)			0.494
Adenocarcinoma	29 (60.4)	26 (63.4)	
Squamous-Cell Carcinoma	14 (29.2)	8 (19.5)	
Large Cell Cancer	2 (4.2)	1 (2.4)	
Others	3 (6.3)	6 (14.6)	
Tumour diameter (mm)	42.94	39.66	0.234
Clinical nodal status (%)			0.286
Negative	22 (48.9)	25 (61.0)	
Positive	23 (51.1)	16 (39.0)	
Pathological nodal status (%)			0.825
Negative	29 (60.4)	26 (65.0)	
Positive	19 (39.6)	14 (35.0)	
Grading (%)			0.051
1	3 (6.5)	4 (10.5)	
2	15 (32.6)	21 (55.3)	
3	28 (60.9)	13 (34.2)	
Nodal stage migration (%)			0.491
Negative to positive	6 (13.3)	3 (7.5)	
Lymphatic vessel invasion (%)			1
No Invasion	43 (89.6)	35 (87.5)	
Invasion	5 (10.4)	5 (12.5)	
Tumour localisation (%)			0.349
Central	15 (31.3)	9 (22.0)	
Peripheral	33 (68.8)	32 (78.0)	
Modality of tissue confirmation (%)			0.083
Mediastinoscopy, EBUS or Bronchoscopy	8 (16.7)	1 (2.4)	
CT-guided Biopsy	30 (62.5)	29 (70.7)	
Both Modalities	10 (20.8)	11 (26.8)	
No (or inconclusive) Tissue Confirmation	0	0

The distribution of tumour grading between group A and group B in the cT2-subgroup showed significantly higher tumour grading in Group A compared to group B (G1 + G2 vs. G3, *p* = 0.017), as can be seen in Table 3.

In this subgroup analysis stage T2 tumours are defined different in the 7th and 8th staging system and might be a confounder in this subgroup. Regarding tumour diameter, there was no difference between the patients with cT2 tumours receiving surgical therapy within 60 days or thereafter. In fact, diameter was slightly larger in group A (42.94 vs. 39.66 mm, *p* = 0.234).

A logistic regression analysis was performed to comprehend the risk reduction of death when being operated in 60 days or less. For patients with a cT2-staged lung tumour the risk of death in the five years following surgical resection can be reduced by approximately 76.3% if they undergo surgery in 60 days or less (OR = 0.0237, 95% CI = 0.059 – 0.945, *p* = 0.041). When performing the same logistic regression analysis for the whole dataset, the result was not significant (*p* = 0.398). See Table 4.

**Table 4:** Logistic regression comparing the 5-year risk of death between group A and B for all patients and the subgroup of cT2 patients.

Patient group	No. of patients	Comparison	OR	95% CI	*p* Value
cT2-staged patients	89	Group A vs. Group B	0.237	0.059—0.945	0.041
All patients	287	Group A vs. Group B	0.722	0.340—1.535	0.398

One reason for a prolonged delay between suspicious imaging and surgery might be waiting times for different modalities of tissue confirmation. When comparing these modalities, Group A showed a significantly lower rate of CT-guided biopsies (89.4% vs. 95.9%; *p* = 0.041). CT-guided biopsy was associated with a significantly longer median delay of 63.00 days in comparison to 51.00 days (*p* = 0.018). Moreover, group B showed a prolonged median time interval from tissue confirmation to operation by 14.50 days (32.00 days vs. 46.50 days; p < 0.001).

**Figure 4:**
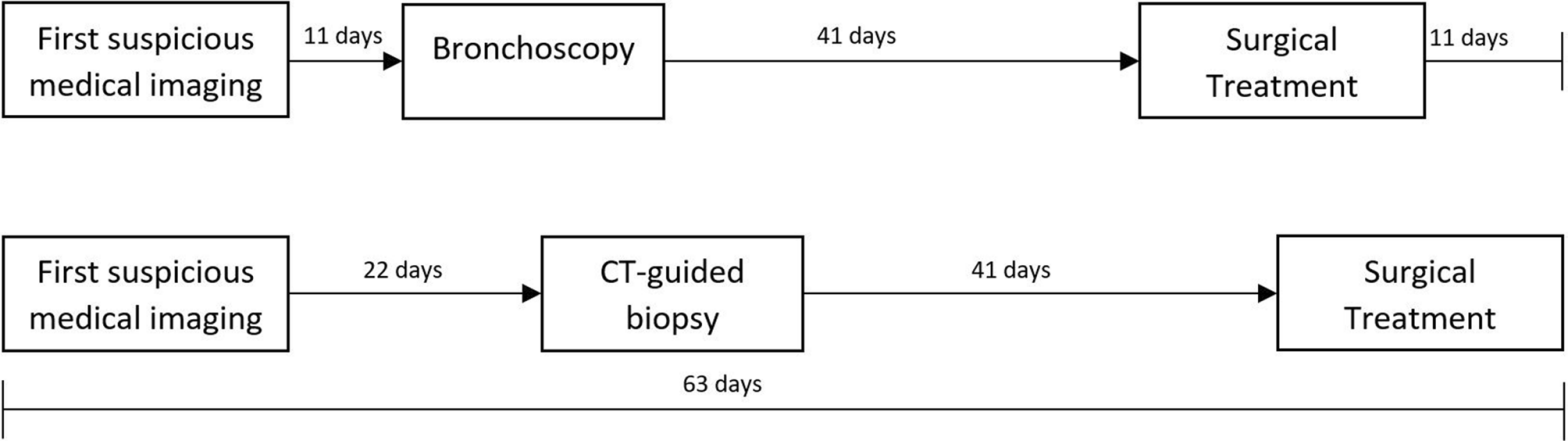
Timeline for delay between first suspicious medical imaging and surgical treatment in stage cT2 patients. CT-guided tissue confirmation adds 11 days of additional delay to the process compared to bronchoscopy alone (*p* = 0.040).

In the cT2-subgroup patients receiving only a bronchoscopy for tissue confirmation showed a 11 day shorter median delay from first suspicious medical imaging to tissue confirmation in comparison to patients receiving a CT-guided biopsy (22.00 days vs. 11.00 days; p 0.040). The subsequent median period from tissue confirmation to operation did not differ significantly (40.50 days vs. 40.50 days; *p* = 0.904). Timelines of delay to surgical treatment are depicted in Fig. 4.

## Discussion

Lung cancer mortality remains high and lung cancer deaths are still on the rise. If curative treatment is possible, it seems logic to start treatment as soon as possible. With this study we tried to answer the question whether delay of surgical treatment in a primary surgical patient group has an impact on oncologic outcome. Median time between first suspicious medical imaging and surgery was 62 days. According to this, the study population was split into two cohorts (group A: delay less than 60 days vs. group B: delay of 61 to 120 days). Patients with a delay of more than 120 days were excluded as this delay is most likely caused by extended follow-up imaging of solitary pulmonary nodules.

In comparison to the median delay of other centres internationally (36.5 – 107 days), this delay is in a comparable range^6–8^. In our cohort, the duration of the delay might be caused by patients, who received their first suspicious medical imaging in the primary care sector and therefore are out of reach for further and streamlined work-up until their first presentation at a secondary care facility.

One possible reason for a prolonged delay to operation at our institution might be tissue confirmation. We consider tissue confirmation as a crucial and vital step before individualized treatment planning in a multidisciplinary tumour board. When bronchoscopy is compared to CT-guided biopsy, the additional delay is about 11 days to schedule a patient for CT-guided biopsy. This delay is mainly caused by adding another step in the process of patient evaluation. Moreover, it involves another specialty with its own subprocesses and its own scheduling policies, which are clearly out of hand of the primary case managers. Scheduling patients in another department for an intervention adds considerable friction to the process. Therefore, careful selection of patients that are suitable for a faster tissue confirmation by bronchoscopy needs to be done, as bronchoscopy can be performed by the primary case manager in the hospital. Ideally, the decision on the method of tissue confirmation is made in a meeting of experts in the different specialties involved in lung cancer work-up.

Comparing group A and group B there was no overall survival benefit for a faster work-up. This may be a result of the high rate of early stage lung cancer patients in our study cohort.

As tumour staging has a strong impact on oncologic outcome, we performed a subgroup analysis of the different cT stages. This approach is a novelty in investigating the impact of delay on survival in lung cancer treatment. There was no survival difference between group A and group B for cT1-staged patients. For cT2-staged patients we could show that surgical resection in 60 days or less offers a significant 5-year survival benefit (85.4% vs. 59.8%, *p* = 0.043) and a 76.3% risk reduction of death in the five years following surgical resection (*p* = 0.041). There was no difference in this cT2-subgroup with regards to other clinical parameters affecting survival, such as tumour diameter, clinical/postoperative lymph node status, nodal stage migration, invasion of lymphatic vessels, tumour grading, age, sex, adjuvant chemotherapy or COPD.

These results indicate that patients with potentially curable lung cancer suffer from dismal survival because of an extended delay to surgery. To our knowledge, this is the first study, which investigates the association of delayed treatment and survival stratified by cT-staging. Available literature only approaches this topic by comparing varying time frames of delay and survival in view of overall UICC lung cancer stages. Looking at these studies some reached comparable results, like Coughlin et al., who showed that UICC stage II NSCLC patients have a significantly decreased survival when the patient work-up from the decision to operate to resection took two months or more^9^. Kasymjanova et al. reached similar results, describing a beneficial effect on survival for early and locoregional stage disease, when the biopsy-to-treatment interval was 30 days or less^10^. In contrast to these results some publications state that there is no relationship between survival and prolonged delay to treatment or even that shorter delay causes reduced survival^11,12^. It has to be noted that these results may be due to advanced-stage lung cancer patients who receive faster treatment because of acute symptom presentation. However, a systematic review by Olsson et al. came to the conclusion that the impact of the timeliness of care remains inconclusive as associated factors need further detailed investigation^13^.

**Figure 5:**
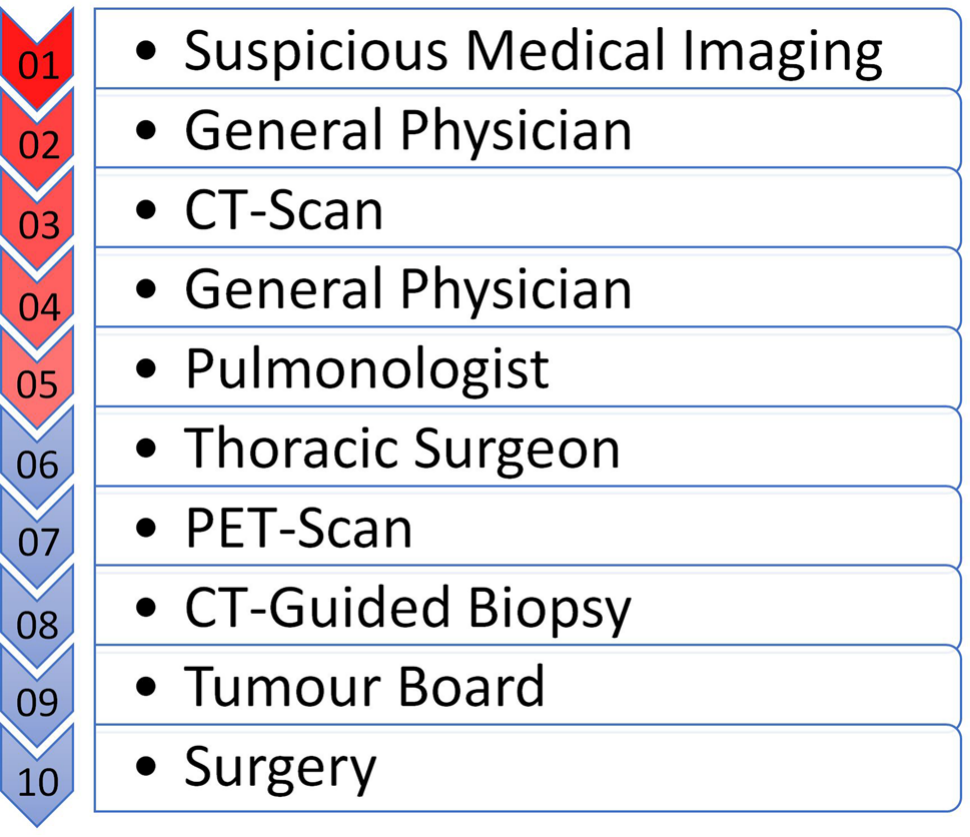
Sub-processes for patient work-up in lung cancer.

Our results suggest the implementation of a faster and more efficient patient pathway to reduce lung cancer mortality. To finally reach primary surgical resection patients go through a multi-step process, which often covers multiple diagnostic measures, referral to specialists and preoperative assessments. (Fig. 5) All of these sub-processes can provide bottlenecks for an efficient patient work-up and further stretch the delay between the first suspicion of lung cancer and treatment^12,14,15^.

Process improvement might be achieved by stringent interdisciplinary work-up and a patient-centred approach. A valid and only slightly disruptive approach to reduce the delay to primary surgery would be to reserve dedicated slots for diagnostic and therapeutic measures for patients with suspected lung cancer. Furthermore a position of a coordinator has to be introduced to streamline and fully organize the patients’ pathway for suspicion of lung cancer to definitive diagnosis or treatment. Through a similar rework, according to the ‘Time to Treat Program’, it was possible to reduce the delay from suspicion to diagnosis from 128 to 20 days in Toronto^16^.

This becomes more important in the light of recently published lung cancer screening trials, which show that lung cancer screening for specific patient groups reduces mortality. Subsequently the rate of operable lung cancer patients will be on the rise, as more early stage cancers will be detected and be subject to primary surgical treatment^17,18^.

From a surgeon’s perspective, these rising numbers of surgical candidates result in the same challenges as many service industries face and can be compared with queuing. In case of surgical units not being able to cope with the rising amount of primary lung cancer resections, it will finally end in progressing patient queuing and therefore will negatively affect lung cancer survival. According to our data, it seems to be justifiable to plan the date of surgical resection in accordance with cT-staging; meaning that it is no necessity for oncologic outcome to resect every lung cancer as fast as possible, but to stratify specific patient groups in fast and normal workup schemes to reduce waiting times for advanced cancer stages. This division might be achieved by establishing a form of risk calculation for categorizing and quantifying the urgency of a fast-track treatment.

In consideration of our results and the emerging of lung cancer screening routines it is urgent to rework our patient pathway to reduce the delay from the first suspicious medical imaging to primary surgery. Efforts need to focus on improving and streamlining patient pathways to shorten the delay until surgical treatment to a minimum. A centralized work-up with professionals in many specialties including radiologists, pulmonologists, pathologists, medical oncologists and thoracic surgeons seems to be essential.

### Limitations

The study design was of retrospective, nonrandomized character. As many patients began their lung cancer journey in the primary care sector, we were not able to reconstruct patient pathways in these cases. We assume that any step in the process of patient work-up causes some delay. To further analyze the impact of these different steps, future studies should investigate the time prior to the first presentation at a secondary care center.

## Conclusions

Delay between diagnosis and treatment of lung cancer results in dismal outcome for specific patient groups. A reason for this delay was found to be histologic tissue confirmation, which is a must in patient work up. Patients receiving a bronchoscopic confirmation were associated with 11-day shorter delay, when compared with CT-guided biopsy.

Through CT-guided biopsy another department is added to the process and thus causing additional friction in patient work up. Efforts need to focus on improving and streamlining patient pathways to shorten the delay until surgical treatment to a minimum. Process improvement might be achieved by stringent interdisciplinary work-up, a patient-centred approach and implementation of standardized work-up-algorithms. Once these algorithms are effective, primary care physicians should make use of this infrastructure and refer patients as early as possible to further reduce the time until final treatment.

## References

[1] Allemani C, Matsuda T, Di Carlo V, Harewood R, Matz M, Nikšić M (2018). Global surveillance of trends in cancer survival 2000–14 (CONCORD-3): analysis of individual records for 37 513 025 patients diagnosed with one of 18 cancers from 322 population-based registries in 71 countries. The Lancet.

[2] Risberg T, Sørbye SW, Norum J, Wist EA (1996). Diagnostic delay causes more psychological distress in female than in male cancer patients. Anticancer. Res..

[3] Robinson E (1984). The fight against the delay in the diagnosis of cancer. Biomed. Pharmacother..

[4] Christensen E (1997). The impact of delayed diagnosis of lung cancer on the stage at the time of operation. Eur. J. Cardiothorac. Surg..

[5] Sobin LH, Gospodarowicz MK, Wittekind C. TNM Classification of Malignant Tumours (ed. Wittekind, C.) 136–146 (Wiley, New York, 2011).

[6] Malalasekera A, Nahm S, Blinman PL, Kao SC, Dhillon HM, Vardy JL. How long is too long? A scoping review of health system delays in lung cancer. *Eur. Respir. Rev.* 2018; 27(149). 10.1183/16000617.0045-2018 PMC9488868 30158277

[7] Didkowska J, Wojciechowska U, Mańczuk M, Łobaszewski J (2016). Lung cancer epidemiology: contemporary and future challenges worldwide. Ann. Transl. Med..

[8] Ferlay J, Soerjomataram I, Ervik M, Dikshit R, Eser S, Mathers C, et al. GLOBOCAN 2012 v1.0: Cancer Incidence and Mortality Worldwide: IARC CancerBase No. 11: International Agency for Research on Cancer; 2013 [cited 2018 Jul 11]. http://globocan.iarc.fr.

[9] Coughlin S, Plourde M, Guidolin K, Fortin D, Frechette E, Malthaner R (2015). Is it safe to wait? The effect of surgical wait time on survival in patients with non-small cell lung cancer. Can. J. Surg..

[10] Kasymjanova G, Small D, Cohen V, Jagoe RT, Batist G, Sateren W (2017). Lung cancer care trajectory at a Canadian centre: an evaluation of how wait times affect clinical outcomes. Curr. Oncol..

[11] Jacobsen MM, Silverstein SC, Quinn M, Waterston LB, Thomas CA, Benneyan JC (2017). Timeliness of access to lung cancer diagnosis and treatment: a scoping literature review. Lung Cancer.

[12] Vinas F, Ben Hassen I, Jabot L, Monnet I, Chouaid C (2016). Delays for diagnosis and treatment of lung cancers: a systematic review. Clin. Respir. J..

[13] Olsson JK, Schultz EM, Gould MK (2009). Timeliness of care in patients with lung cancer: a systematic review. Thorax.

[14] Kim JOA, Davis F, Butts C, Winget M (2016). Waiting time intervals for non-small cell lung cancer diagnosis and treatment in alberta: quantification of intervals and identification of risk factors associated with delays. Clin. Oncol. (R Coll. Radiol.).

[15] Maiga AW, Deppen SA, Pinkerman R, Callaway-Lane C, Massion PP, Dittus RS (2017). Timeliness of care and lung cancer tumor-stage progression: how long can we wait?. Ann. Thorac. Surg..

[16] Lo DS, Zeldin RA, Skrastins R, Fraser IM, Newman H, Monavvari A (2007). Time to treat: a system redesign focusing on decreasing the time from suspicion of lung cancer to diagnosis. J. Thorac. Oncol..

[17] Aberle DR, Adams AM, Berg CD, Black WC, Clapp JD, Fagerstrom RM (2011). Reduced lung-cancer mortality with low-dose computed tomographic screening. N. Engl. J. Med..

[18] De Koning H, van der Aalst C, ten Haaf K, Oudkerk M (2018). PL02.05 effects of volume CT lung cancer screening: mortality results of the NELSON randomised-controlled population based trial. J. Thorac. Oncol..

